# Use of a commercial enzyme immunoassay to monitor dengue virus replication in cultured cells

**DOI:** 10.1186/1743-422X-5-51

**Published:** 2008-04-25

**Authors:** Juan E Ludert, Clemente Mosso, Ivonne Ceballos-Olvera, Rosa M del Angel

**Affiliations:** 1 Departamento de Patología Experimental, Centro de Investigación y de Estudios Avanzados del I.P.N., Mexico City, Mexico

## Abstract

Current methods for dengue virus quantitation are either time consuming, technically demanding or costly. As an alternative, the commercial enzyme immunoassay Platelia™ Dengue NS1 AG (Bio-Rad Laboratories) was used to monitor semiquantitatively dengue virus replication in cultured cells. The presence of NS1 protein was evaluated in supernatants from Vero and C6/36 HT cells infected with dengue virus. The amount of NS1 detected in the supernatants of infected cells was proportional to the initial MOI used and to the time of post infection harvest. This immunoassay was also able to detect the presence of NS1 in the supernatants of infected human macrophages. Inhibition of dengue virus replication in C6/36 HT cells treated with lysosomotropic drugs was readily monitored with the use of this assay. These results suggest that the Platelia™ Dengue NS1 AG kit can be used as a fast and reliable surrogate method for the relative quantitation of dengue virus replication in cultured cells.

## Background

Dengue is one of the most important arthropod-borne viral diseases in tropical and subtropical areas around the world and represents a serious public health in several countries of America, Asia and Africa. Last year, only in the Americas more than 800,000 cases of dengue fever, the less severe clinical form of dengue infection, and more than 25,000 cases of dengue hemorrhagic fever, the most severe form of dengue syndrome, occurred [1]. Although during the past years, the incidence of dengue has grown in endemic areas, a specific treatment or vaccines are not yet available.

The four antigenically related serotypes of dengue virus (DEN): DEN1, DEN2, DEN3 and DEN4, members of the *Flavivirus *genus (family *Flaviviridae*), are transmitted to humans by *Aedes aegypti *mosquitoes. DEN is an enveloped virus of 50 nm in diameter and contains a single strand and positive-polarity RNA as genome of about 10.7 kb [2]. DEN genome encodes for three structural proteins (envelope glycoprotein, E; membrane, M; and capsid, C) and for seven non-structural proteins (NS1, NS2a, NS2b, NS3, NS4a, NS4b and NS5). E protein is the major structural protein exposed on the surface of the particle.

The detection of antibodies directed against E protein is the most common technique to detect DEN infection in diagnostic test, such as the capture enzyme-linked immunoadsorbent assay MAC-ELISA. In addition, some other non-serological techniques such as virus isolation and reverse transcriptase-polymerase chain reaction (RT-PCR) have been used to demonstrate completely the presence of viral particles in sera from infected patients collected during the acute phase of the disease as well as in supernatants of infected cells. Virus isolation and titration are also very useful tools for the quantitation of DEN. However, both procedures are expensive and time-consuming [3,4]. On the other hand, real time PCR or competitive RT-PCR are also very helpful techniques to quantify viral RNA in human sera as well as in infected cells [5-9]. However, for molecular methodologies, RNA isolation, expensive reagents, specialized equipment and internal controls are required. Pitfalls in the standard techniques used for the quantitation of DEN have prompted the search of alternative semiquatitative methods. Recently, a flow cytometry-based assay and a fluorescent focus assay for flavivirus quantitation have been reported [10,11].

An attractive alternative for the quantitation of viral infection efficiency is to measure the amount of a particular viral protein. NS1 is a highly conserved nonstructural glycoprotein of DEN, which exists predominantly in a dimeric form, which is associated with intracellular and cell surface membranes [12]. Although the precise role of NS1 protein in the flavivirus life cycle remains unclear, NS1 dimers have been shown to interact with other non-structural viral proteins and through this association, with the viral RNA. Then, NS1 protein may be involved in assembly of the viral replicase complex and its localization to cytoplasmic membranes [13-16]. NS1 protein is secreted from infected cells as a soluble, detergent-labile hexamer [17]. Furthermore, it has been demonstrated that NS1 antigen is present in the sera of acute-phase infected patients, and in DEN infected cell cultures, and that supernatant levels of NS1 protein correlate with infectious titers [18,19]. Recently, a commercial enzyme immunoassay, Platelia™ Dengue NS1 AG (Bio-Rad Laboratories), was developed for the detection of NS1 antigen in human serum or plasma. This assay has been reported to be useful for the diagnosis of DEN infection, particularly during the early-acute-phase [20,21].

Based on the fact that NS1 protein in infected cell cultures, as well as in humans, is produced and secreted and that the amount of NS1 protein correlates with the viral replication efficiency, in this study we evaluated the use of Platelia™ Dengue NS1 AG as a surrogate method to monitor semiquantitatively DEN replication in cultured cells. Our results indicate that this commercial assay could be a useful method to monitor differences in DEN replication under different experimental conditions in a short time period (less than three hours).

## Results

### The amount of NS1 protein secreted by DEN infected cells correlates with the multiplicity of infection

**Figure 1:**
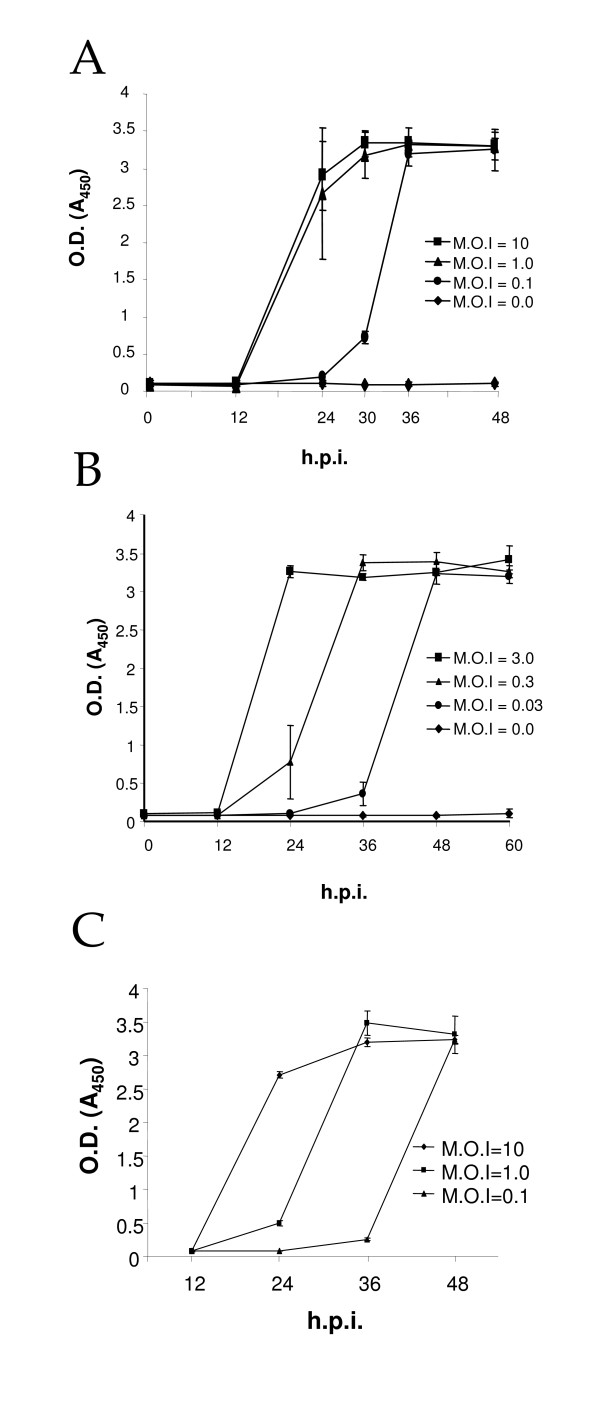
**Detection of NS1 protein in supernatant media from cells infected with dengue virus**. Vero (A) or C3/36 HT cells (B) were infected with dengue 2 virus at different MOIs. At different times post-infection, the media were collected and tested for the presence of NS1 protein with Platelia™ Dengue NS1 Ag kit. (C) Ten micro liters of the supernatant media collected from the experiments shown in (A) were mixed with 40 μl of diluent buffer provided with the kit and used to measure NS1 protein levels with Platelia™ Dengue NS1 Ag kit. Points in (A) and (B) are mean values ± SD of three independent experiments.

To evaluate the convenience of the Platelia™ Dengue NS1 AG enzyme immunoassay for the quantitation of NS1 protein in the culture medium of infected cells, sub-confluent monolayers of Vero and C6/36 HT cells were infected with different MOIs of DEN2 and the amount of NS1 protein released to the culture media was evaluated up to 48 hours post infection (hpi) for Vero cells and up to 60 hpi for C6/36 HT cells using Platelia™ Dengue NS1 AG kit. In Vero cells, NS1 protein was detected after 12 hpi and reached a plateau at 36 hpi for all three MOIs used (Figure 1A). At 24 hpi, obvious differences in the level of NS1 were observed between the cells infected with MOIs of 0.1 and 1. However, these differences were not clear between Vero cells infected at a MOI of 1 and 10.

When NS1 levels were measured in supernatants from infected C6/36 HT cells, we could observe that the kinetics at the three MOI used were very similar, but showed a 12 h lag between them. At MOI of 3 the production of NS1 protein reached a plateau 24 h after infection, while at MOI of 0.3 the plateau was reached at 36 h after infection, and at the lowest MOI used (0.03) the plateau was reached at 48 h after infection (Figure 1B). Of note, even though three independent experiments were carried out with each cell line, low standard deviations were obtained for most of the points of the curves, indicating high reproducibility for the method. Moreover, OD readings obtained from samples run in triplicate were quite similar supporting our previous statement.

One plausible explanation for the failure to detect clear differences in NS1 levels in the supernatants obtained from Vero cells inoculated with MOIs of 1 and 10, could be the high sensitivity of the kit and the saturation of the system. To address this possibility, the level of NS1 was monitored in 10 μl aliquots of supernatant of infected cells instead of the 50 μl used previously. Upon dilution of the supernatant, a clear correlation between the MOI used to infect the Vero cells and the level of NS1 produced was observed (Figure 1C). At 24 hpi, an obvious difference in the level of NS1 was observed between the cells infected with MOIs of 1 and 10. Furthermore, the kinetics at the three MOIs used were very similar, but showed a 12 h lag between them resembling the kinetics observed with C6/36 HT cells (Figure 1B). These results suggest that although the system is saturable, dilutions of the supernatant permit to reveal differences in the NS1 levels among samples. Thus, although Platelia™ Dengue NS1 AG was not designed to be a quantitative assay, it is evident that the OD readings obtained from the ELISA could reflect the amount of NS1 protein produced as a function of time and MOI.

**Figure 2:**
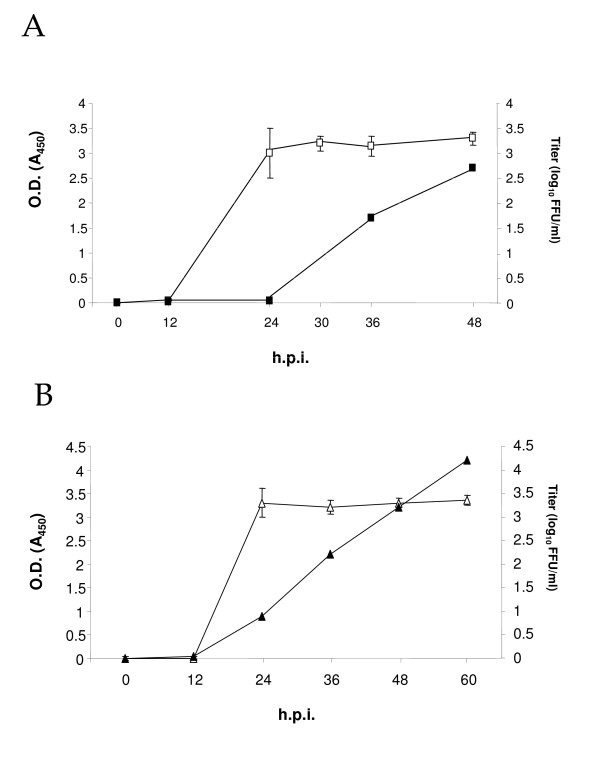
**Correlation between focus forming units and NS1 levels in Vero (A) and C6/36 HT (B) cells**. Supernatant from Vero (A) and C6/36 HT cells (B) infected at a MOI of 10 and 3 respectively were collected and used for: measurement of NS1 protein levels with Platelia™ Dengue NS1 Ag kit (open symbols) and for focus forming units (FFU) calculation (solid symbols). Points are mean values of three independent experiments.

A correlation between virus yield and level of secreted NS1 has been described in other studies [13-16,19]. To study the relationship between NS1 levels detected by Platelia™ Dengue NS1 AG and virus yield, the amount of virus presents in the supernatants of Vero and C6/36 HT cells infected with the maximum MOI, 10 and 3 respectively, were titrated by focus forming unit assay. Titer obtained by plaques and focus forming units assays are very similar [10]. In Vero cells, infectious particles were first detected after 36 hpi and increased one log at 48 hpi (Figure 2A). For C6/36 HT cells infected with MOI of 3, virus particles were first detected after 24 hpi, and increased exponentially at 36, 48 and 60 hpi (Figure 2B).

### NS1 protein is detectable in the culture media from infected macrophages

To test the convenience of the Platelia™ Dengue NS1 AG for the detection of NS1 protein in DEN2 infected primary cultures, human peripheral blood CD14 cells isolated from three healthy donors by anti-CD14 antibody affinity column, were cultured for 7 days in RPMI 1640 medium to generate mature macrophages. Macrophages were infected with a MOI of 3 and the infection was permitted for 24 and 48 h. Platelia™ Dengue NS1 AG permits to monitor DEN replication in the supernatant of primary cultured cells after 48 hrs, although the OD readings varied widely among donors (0.65, 1.66 and 3.15). Supernatants collected at 0 and 24 hpi tested negative for NS1 protein.

### NS1 protein production can be used to determine the effect of certain compounds or drugs in viral infection

**Figure 3:**
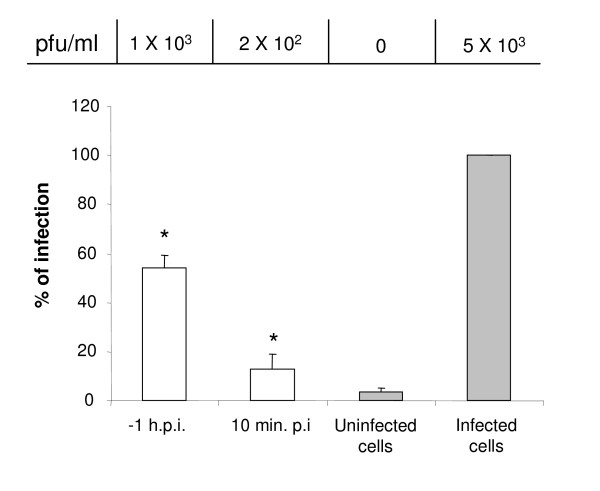
**Detection of NS1 protein in supernatant media from cells treated with lysosomotropic agents**. C6/36 HT cells infected with dengue 2 virus at MOI of 10 were either treated for 1 h, before infection (-1 hpi) or 10 min post-infection (10 mpi) with 50 mM NH_4_Cl (open bars). As a control, non drug treated cells, either mock infected or infected, were run in parallel (grey bars). At 24 hpi the supernatant media were collected and tested for the presence of NS1 protein with Platelia™ Dengue NS1 Ag kit. Results are expressed as the percentage of the OD (A_450_) obtained in non-drug treated, infected cells. Results are mean values ± SD of three independent experiments. The decrease in NS1 production in drug treated cells was statistically significant compared to the control condition (* *p *< 0.05). Titers for each condition obtained by plaque assay and expressed as PFU/ml are shown on the top of the bars.

In an effort to prove that the kit could be used to monitor infection efficiency under experimental conditions, an infection inhibition assay was performed. It has been described that the fusion mediated by the E protein requires the low pH present in internal vesicles [22]. Thus, it was expected that compounds such as NH_4_Cl would inhibit DEN fusion and infection in C6/36 HT cells and that such inhibition could be monitored by measuring the amount of NS1 protein present in 10 μl of the media with the Platelia™ Dengue NS1 AG kit. C6/36 HT cells were incubated for 1 h with 50 mM of NH_4_Cl, 1 h before or 10 min after the infection with DEN2 at MOI of 10. The OD of the untreated infected cells was considered as 100% and treated samples were referred as a percentage of the control. Figure 3 shows that the production of NS1 protein was inhibited up to 50% when NH_4_Cl was added 1 h before infection while a 90% inhibition was observed when the NH_4_Cl was added 10 min after infection. Furthermore, the differences observed between treated and untreated cells in the production of NS1 showed a relationship with differences in the viral titer, measured using a plaque forming units assay, obtained in the supernatant of the treated and untreated cells (Figure 3).

## Discussion

Different techniques have been used to quantify DEN infection in human sera and in medium from infected cells. Titration by plaque assays is considered the gold standard, but additional techniques which include real time RT-PCR, competitive RT-PCR or flow cytometry using anti-E protein antibodies have also been used for that purpose. Although all of them are useful methods, they may be expensive, time-consuming and require specialized equipment [3,4,6,8-11]. Therefore, we decided to evaluate the detection of DEN NS1 protein with the Platelia™ Dengue NS1 AG kit as a surrogate, semiquantitative method, to monitor viral replication efficiency. The Platelia™ Dengue NS1 AG assay is a one step microplate enzyme immunoassay that has been reported to be simple, rapid, subject to quality assurance and robust [20,21]. Although the precise roles of NS1 protein in the flavivirus life cycle remain unclear, its presence correlates with viral replication efficiency [13-16,19]. Our results with Vero and C6/36 HT cells confirm that the secretion of NS1 shows a relationship with viral replication. Moreover, given the high sensitivity of the kit, when the amount of NS1 in 50 μl of supernatant are enough to saturate the system, a low level of viral particles, measured by FFU, were detected. This observation indicates that Platelia™ Dengue NS1 AG may actually be used to detect dengue virus infections at early times, when levels of mature virions are still too low to be reliable measured by FFU assays. The results with the grow curves obtained in DEN infected Vero as well as in C6/36 HT cells using different MOIs clearly suggest that Platelia™ Dengue NS1 AG assay can be used as a fast surrogate marker to monitor semiquantitatively viral infection in both cell types. This method may also be a good tool to monitor DEN infection in primary culture cells such as macrophages. However, relative quantitation may be difficult due to differences in the number of macrophages actually differentiated and infected from each donor. Furthermore, the amount of NS1 release from infected macrophages may also be affected by individual host cell factors.

Interestingly, several reports indicate that DEN NS1 protein is secreted from mammalian cells but not from mosquito cells [17,18]. In contrast, we found high levels of NS1 protein in the supernatant media of infected C6/36 HT after 24 hpi regardless of the MOI used. Secretion of NS1 protein to the culture medium from DEN infected C6/36 cells or from insect cells expressing recombinant dengue virus NS1 protein has been observed by others [23,24]. It has been proposed that proper processing of N-glycans appears to be essential for the maturation, transport and secretion of NS1 protein from infected cells [17]. Thus, discrepancies in the detection of NS1 protein in supernatant from C6/36 infected cells most likely obey to heterogeneities in the glycoconjugates biosynthesis among the C6/36 cell lines employed [17,23].

On the other hand, the experiments performed with lysosomotropic drugs, in which inhibition of DEN infection by the effect of such agents could be easily monitored, showed as a proof of principle, that Platelia™ Dengue NS1 AG assay could be used as an excellent tool for fast and reliable relative quantitation of dengue virus replication in cultured cells. Moreover, the reduction in the level of NS1 protein released from treated cells correlates with a reduction in plaque forming units detected in these supernatants by plaque assay, supporting our previous conclusion. The lesser inhibitory effect upon the infection observed when the cells were pretreated with NH_4_Cl than when the drug was administered 10 min after the inoculum must likely reflects the capacity of the cells to extrude the drug and compensate the endosomal pH.

Because the current version of the Platelia™ Dengue NS1 AG assay was primarily designed to discern qualitatively between DEN infected and no infected patients, it is not a quantitative test. Given the sensitivity of the test, maximum OD values are often obtained. Furthermore, no information of the limit of NS1 amount detected by the kit is provided by the manufacturer. Thus, proper quantitation will require serial dilutions of the samples and comparison with standard curves derived from titration series of purified NS1 protein corresponding to the DEN serotype being used [18,19]. However, for detection of NS1 protein in cell culture media, this limitation can be overcome partially by using diluted amounts of culture media as shown in Figure 1C or by testing serial dilutions of the supernatant to reach a detection limit (data not shown). The low standard deviations observed in most of the points of the graphics presented clearly indicate that the results are quite reproducible. However to ensure comparability, we recommend testing all samples and controls in the same plate, along with the controls included in the kit.

In conclusion, our data suggest that the Platelia™ Dengue NS1 AG assay can be use as a surrogate, easy and fast method for the semiquantitation of DEN in cultured cells. Reliable levels of NS1 protein for quantitation are usually reached in the cell supernatant after 24 hpi and the analysis can be carried out in less than 3 hours. In addition, the low background levels obtained with mock infected cells allowed easy discrimination between positive and negative samples. However, DEN quantitation continues to pose difficulties and the development of a quantitative test based on NS1 protein detection is highly desirable.

## Materials and methods

### Cells

Vero cells were grown in Dulbecco's modified eagle medium (D-MEM Advanced, Gibco-BRL) supplemented with 8% fetal calf serum (Gibco-BRL), 2 mM L-glutamine, 1.5 g/l sodium bicarbonate, 50 U/ml of penicillin and 50 μg/ml of streptomycin. BHK21 cells were grown in minimal essential medium (MEM, Gibco-BRL) supplemented with 10% fetal calf serum (GIBCO-BRL), 2 g/l sodium bicarbonate, 50 U/ml of penicillin and 50 μg/ml of streptomycin. C6/36 HT cells (derived from the C6/36 *Aedes albopictus *cells but adapted to grow at 34°C) kindly provided by Dr. Goro Kuno, CDC, Puerto Rico, were grown in MEM, supplemented with 7% fetal calf serum, nonessential aminoacids, vitamins, 0.370 g/l sodium bicarbonate, 50 U/ml of penicillin and 50 μg/ml of streptomycin at 34°C.

Human peripheral blood mononuclear cells (PBMCs) were purified from peripheral blood obtained from healthy donors by Ficoll-Hypaque (Pharmacia) density gradient centrifugation. Monocytes were purified from human PBMCs using MACS CD14 microbeads (Miltenyi Biotec) according to the manufacturer's recommendations. Purity was checked by FACScan analysis by staining the cells with an anti-CD14 antibody conjugated with FITC (Sigma-Aldrich). Macrophages were obtained by incubation of adherent cells in 96 well plates (1 × 10^5 ^cells/well) with RPMI 1640 media supplemented with 5% fetal calf serum for approximately 7 days.

### Viruses

DEN serotype 2 (DEN2), strain 16681, generously provided by Dr. Richard Kinney (CDC, Fort Collins, CO), was propagated in suckling mice brain as previously described [25]. Virus titers in mice brain homogenates were determined by plaque assay.

### Virus inoculation

Twenty four-well plates were seeded with either Vero or C6/36 HT cells and incubated at 37°C in 5% CO_2 _atmosphere or at 34°C respectively, until confluence was reached. After washing the monolayers once with PBS, cells were inoculated with DEN at the appropriate multiplicity of infection (MOI) in a final volume of 0.2 ml. Given the higher capacity of DEN to infect mosquito cells compared to Vero cells, C6/36 HT cells were infected at MOIs of 3, 0.3 and 0.03 while Vero at MOIs of 10, 1 and 0.1. The virus was left to absorb for 1 h. After absorption, the inoculum was removed by aspiration, the monolayers were washed once with PBS and 1.0 ml of medium was added. At appropriate times post infection, the supernatant media were collected and stored at -20°C for virus titration by focus forming units assay and to monitor the presence of NS1 protein with the Platelia™ dengue NS1 Ag kit.

Macrophages, grown in 96-well plates, were infected with DEN2 at MOI of 3, or mock infected, for 1 hour at 37°C. After inoculation, cells were washed twice with medium, 0.1 ml of fresh RPMI 1640 medium added per well and infection was allowed to proceed for 0, 24 or 48 h at 37°C. The supernatant media were stored at -20°C until tested for the presence of NS1 protein.

### Plaque assay

Dengue virus titers were determined by plaque assay on confluent monolayers of BHK-21 cells grown in 24-well plates and cultured in MEM-supplemented with 10% fetal calf serum as previously described [26]. Briefly, when the adherent BHK-21 cells reached 80 to 90% confluence 0.25 ml aliquots of mice brain homogenates or cells supernatants from dengue virus-infected C6/36 HT cells were inoculated at ten-fold serial dilutions from 10^-1 ^to 10^6^. After 4 hrs of viral adsorption, the BHK-21 cell monolayers were overlaid with MEM containing 3% carboximethil-cellulose (Sigma), 0.5% fetal calf serum and 2 mM L-glutamine. The cultures were incubated at 37°C for six days and then counted for plaque formation after fixation with 10% formalin and staining 0.5% naphtol-blue-black (Sigma).

### Immunohistochemical focus assay

Focus forming assays were carried out as described by Payne et al. [11] with modifications. Confluent monolayers of Vero cells grown in 24-well plates were inoculated with 10-fold serial dilutions of supernatant media in a final volume of 0.25 ml. Viral absorption was allowed for 1 h at 37°C. An overlay of MEM, 5% fetal calf serum and 1% carboxymethyl-cellulose (Sigma-Aldrich Co., St. Louis, MO) was added after the inoculum was removed and cell monolayers were washed once with Hank's solution. The overlay was removed at 72 hpi, and cells were fixed for 20 min at room temperature with ice-cold absolute methanol. DEN foci were labeled with a mixture of anti-DEN E (4G2) and pre-M proteins (2H2) Mabs (a kind gift of Dr. Ferdinando Liprandi, Instituto Venezolano de Investigaciones Científicas, Caracas) and a secondary antibody conjugated to alkaline phosphatase. Foci were stained using a combination of 5-bromo-4-chloro-3'-indolylphosphate *p-*toluidine salt and nitro-blue tetrazolium chloride as substrate (BCIP/NBT kit; Invitrogen, Carlsbad, CA) and counted on a light box with the aid of a 10× magnifying glass.

### Lysosomotropic drug treatment

To inhibit the infectious entry of DEN2, C6/36 HT cells grown in 12-well plates were either pretreated with NH_4_Cl (50 mM, final concentration) for 1 h at 34°C before infection or the drug was added 10 min post infection and left for 1 h. Cells were infected at a MOI of 10 in a final volume of 0.5 ml for 1 h at 34°C. After inoculation, cells were washed twice with PBS to remove unbound virus and once with acid glycine (pH 2.8) to inactivate viruses that failed to enter. After 2 additional washes with PBS, 1.0 ml of medium was added to each well and cells incubated at 34°C for 24 h. At this time, the media were collected and titrated by plaque assay and tested for the presence of NS1 protein. Mock infected and virus infected, non-drug treated cells, were run in parallel as negative and positive controls respectively. Differences in NS1 levels between drug treated and non-treated cells were tested for significance by the Student's *t *test.

### PLATELIA™ assay

The Platelia™ Dengue NS1 Ag is a one step sandwich format microplate enzyme immunoassay for the qualitative or semi-quantitative detection of dengue NS1 antigen in human sera or plasma. The test uses murine monoclonal antibodies for capture and revelation. The assay was carried out following the procedure indicated by the manufacturer, except when indicated. In brief, reagents and samples were left to warm at room temperature and 50 μl of supernatant media were incubated directly and simultaneously with the conjugate for 90 min at 37°C within the microplate wells sensitized with monoclonal antibody anti-NS1. For particular experiments, only 10 μl of supernatant media were used. After a washing step, the presence of immune complexes was detected by a color development reaction using 3, 3', 5, 5' tetramethylbenzydine as a substrate. The color development reaction was stopped after 30 min incubation at room temperature by the addition of an acid solution. Finally, the optical density (OD) was determined at 450 nm using an automatic ELISA plate reader (Multiskan EX, Labsystems). Negative, positive and cut-off control reagents provided with the kit were run each time for validation of the assay. For simplicity, results are expressed directly as OD and not as a ratio OD sample/OD cut-off as recommended by the manufacturers. However, there are no differences if the results are expressed either way.

## Competing interests

The authors declare that they have no competing interests.

## Authors' contributions

JEL performed the experiments presented in figures 1 and 2. CM performed the experiments presented in figure 3. IC–O performed the experiments with human macrophages. JEL and RMDA conceived of the study, participated in study design and jointly prepared the manuscript. All the authors read and approved the final manuscript.
